# Dynamic Functional Connectivity Changes in Cortical and Cortico‐Striatal Strokes in Mice

**DOI:** 10.1002/cns.71114

**Published:** 2026-08-27

**Authors:** Fatemeh S. N. Mahani, Michael Diedenhofen, Claudia Green, Dirk Wiedermann, Gereon R. Fink, Mathias Hoehn, Markus Aswendt

**Affiliations:** ^1^ Cognitive Neuroscience, Institute of Neuroscience and Medicine (INM‐3), Research Centre Juelich Juelich Germany; ^2^ Department of Neurology, Faculty of Medicine University Hospital Cologne, University of Cologne Cologne Germany; ^3^ Department of Neurology, Experimental Neurology Section Goethe University Frankfurt and University Hospital Frankfurt am Main Germany; ^4^ In‐Vivo‐NMR Laboratory Max Planck Institute for Metabolism Research Cologne Germany

**Keywords:** functional brain network analysis, interhemispheric connectivity, MCAO, photothrombosis, resting‐state fMRI, rodents, seed strength, sensorimotor network

## Abstract

**Aims:**

To determine how lesion size and location shape longitudinal functional connectivity (FC) changes after stroke.

**Methods:**

Adult male mice underwent atlas‐based resting‐state fMRI at baseline and 1, 2, and 4 weeks after either a small photothrombotic cortical stroke (*N* = 25) or a larger transient cortico‐striatal MCAO stroke (*N* = 6). FC was quantified across 98 atlas regions, focusing on sensorimotor cortex, striatum, and thalamus, including intra‐ and inter‐hemispheric connectivity matrices and regional seed strength.

**Results:**

Cortical stroke caused widespread hyperconnectivity at Weeks 1–2, with about 90% of connections increased, followed by partial normalization by Week 4. This effect declined most strongly in the ischemic hemisphere and remained more sustained contralesionally. In contrast, cortico‐striatal stroke induced global hypoconnectivity at Week 1, with more than 90% of connections decreased, a modest and heterogeneous shift toward baseline at Week 2, and widespread decreases persisting at Week 4. A subset of sensorimotor connections showing opposite changes in the two models robustly separated groups at all post‐stroke time points. Regional lesion involvement scaled with the magnitude of baseline‐referenced FC alterations.

**Conclusion:**

Lesion topography drives distinct longitudinal FC trajectories after stroke and may help define network biomarkers and optimal windows for targeted interventions.

## Graphical abstract

Atlas‐based resting‐state fMRI revealed distinct post‐stroke FC trajectories in mice: small cortical lesions caused widespread hyperconnectivity at Weeks 1–2 with partial normalization by Week 4, whereas larger cortico‐striatal lesions caused early hypoconnectivity and persistent decreases. Lesion topography and extent shaped the magnitude of FC disruption.

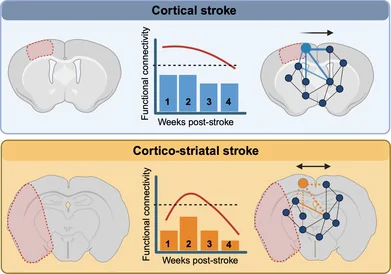

## 1 Introduction

Due to the chronic nature of the most significant stroke symptoms in the sensorimotor and cognitive domains, stroke is the leading cause of acquired disabilities in adults and causes a major life‐complicating and economic burden for patients and society [1]. While acute reperfusion therapies such as thrombolysis and thrombectomy limit initial brain injury, no clinical therapy currently targets the biological mechanisms that support spontaneous functional recovery processes [2]. In functional magnetic resonance imaging (fMRI) studies, the brain network dynamics after stroke have been attributed to functional deficits and to the prediction of recovery potential [3]. Primarily, the interhemispheric functional connectivity (FC) between primary motor areas is strongly associated with motor recovery [4]. The use of FC analysis in neurological disease models in rats and mice has exponentially increased, and the method has been further advanced in terms of the MR sequence, anesthesia, and post‐processing [5, 6]. However, only a limited number of experimental stroke studies used (rs‐)fMRI to correlate FC to behavioral deficits and recovery after stroke [6]. Similar to findings in humans, interhemispheric connections acutely decline after large cortico‐striatal strokes or small cortical strokes, followed by partial normalization toward pre‐stroke levels. This normalization is associated with functional improvement [7, 8], a process that can be enhanced by cell therapy or neuromodulation [9, 10]. However, these studies fall short of fully characterizing longitudinal FC changes and their relationships with lesion size and location, as well as with motor recovery, as they typically focus on either specific connections or global network alterations. Experimental stroke models differ in lesion size, lesion topography, affected circuitry, and ischemic pathophysiology. These differences are functionally relevant because post‐stroke recovery depends on lesion location and volume, damage to fiber tracts, peri‐lesional plasticity, and large‐scale bihemispheric network reorganization [11]. Photothrombotic stroke produces a focal permanent cortical lesion, whereas transient middle cerebral artery occlusion (MCAO) involves ischemia–reperfusion injury and a prominent surrounding penumbra, resulting in distinct biological injury mechanisms [12]. Motivated by findings in human studies, focal cortical lesions are expected to mainly affect local cortical and cortico‐cortical networks, while cortico‐striatal lesions additionally involve subcortical circuits, including cortico‐striatal and thalamo‐cortical pathways [13]. However, a direct comparison of the two stroke models is missing. Since rs‐fMRI detects comparable stroke‐induced network changes in mice and humans [14], and since cortical and cortico‐striatal stroke models show distinct structural connectivity trajectories [15], such a comparison would allow testing whether lesion topography and subcortical involvement determine distinct longitudinal FC trajectories after stroke.

Here, we investigated longitudinal resting‐state FC changes within and between sensorimotor, striatal, and thalamic regions after permanent cortical or transient cortico‐striatal stroke in mice, as these networks are central to post‐stroke recovery [16, 17]. Over 4 weeks, we quantified FC and seed strength to map intra‐ and interhemispheric network reorganization in both stroke models.

## 2 Methods

### 2.1 Experimental Design

Mice were kept in a 12‐h light/dark cycle with access to food and water. Longitudinal in vivo MRI data were obtained from studies reported previously [9, 18, 19]. Two stroke models were analyzed: (i) permanent cortical photothrombosis in 25 adult male C57Bl/6J mice (8–12 weeks, 25–30 g; The Jackson Laboratory), and (ii) transient cortico‐striatal MCAO in 6 adult male NMRI‐nu mice (12–14 weeks, 32–37 g; Janvier Labs). Photothrombosis was induced after Rose Bengal injection by focal cortical illumination, producing localized thrombus formation as described previously [18]. MCAO was induced by insertion of a silicon‐coated filament into the internal carotid artery for 30 min followed by reperfusion [9].

### 2.2 MRI Acquisition and Processing

MRI was performed on a 9.4 T Bruker BioSpec 94/20USR system with cryo‐coil and ParaVision 6.0.1. Mice were initially anesthetized with isoflurane (2%–3% in 70/30 N2/O_2_), positioned in the animal cradle, and monitored for respiration and body temperature throughout the scan. High‐resolution T2‐weighted (T2w) images were acquired, followed by resting‐state fMRI under medetomidine sedation to assess FC (Table 1). AIDAmri v1.3 [20], available from https://github.com/Aswendt‐Lab/AIDAmri was used for processing and registration of T2w and rs‐fMRI data with the Allen Mouse Brain Atlas (CCF v3.0) ‐ Allen Institute for Brain Science (2004), available from mouse.brain‐map.org [21] (for details see Appendix S1).

**TABLE 1:** MRI parameters listed for T2‐weighted and EPI‐based sequences.

Study	Sequence	TR (ms)	TE (ms)	Flip angle	Matrix × slices	Repetitions/volumes	Scan time (min)	Voxel (μm^3^)	FOV (mm^2^)
PT	Turbo‐RARE	5500	32.5	90	256 × 256 × 48	NA	5.9	68.4 × 68.4 × 300	17.5 × 17.5
	rs‐fMRI	1420	18	90	96 × 96 × 20	355	~8.4	182 × 182 × 500	17.5 × 17.5
MCAO	Turbo‐RARE	5500	32.5	180	256 × 256 × 48	NA	5.9	68.4 × 68.4 × 200	17.5 × 17.5
	rs‐fMRI	2840	18	80	96 × 96 × 16	105	5	182 × 182 × 500	17.5 × 17.5

**FIGURE 1:**
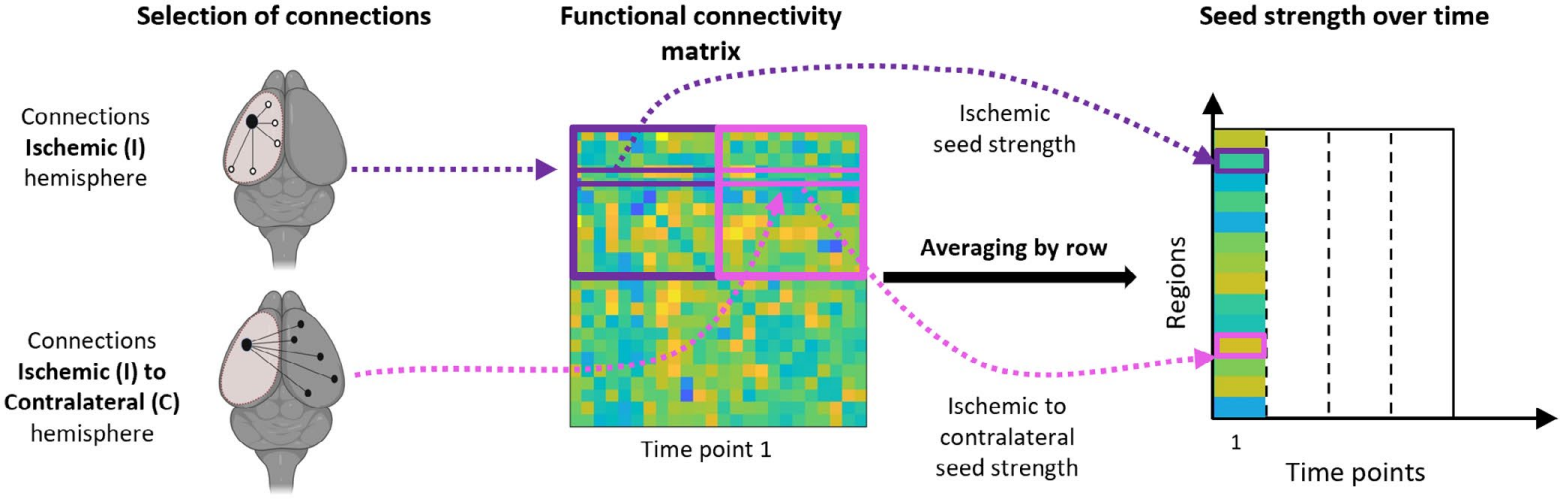
Seed strength calculation. The analysis distinguishes intra‐ and inter‐hemispheric connections to evaluate the average connectivity of each region. Seed strength was derived from the FC matrix, which quantifies the connectivity between all pairs of brain regions. For each region, intra‐hemispheric (purple box) and inter‐hemispheric (pink box) connections were extracted and averaged across animals to obtain a single seed strength value per region. This process was repeated across multiple time points, generating a time‐series plot.

**FIGURE 2:**
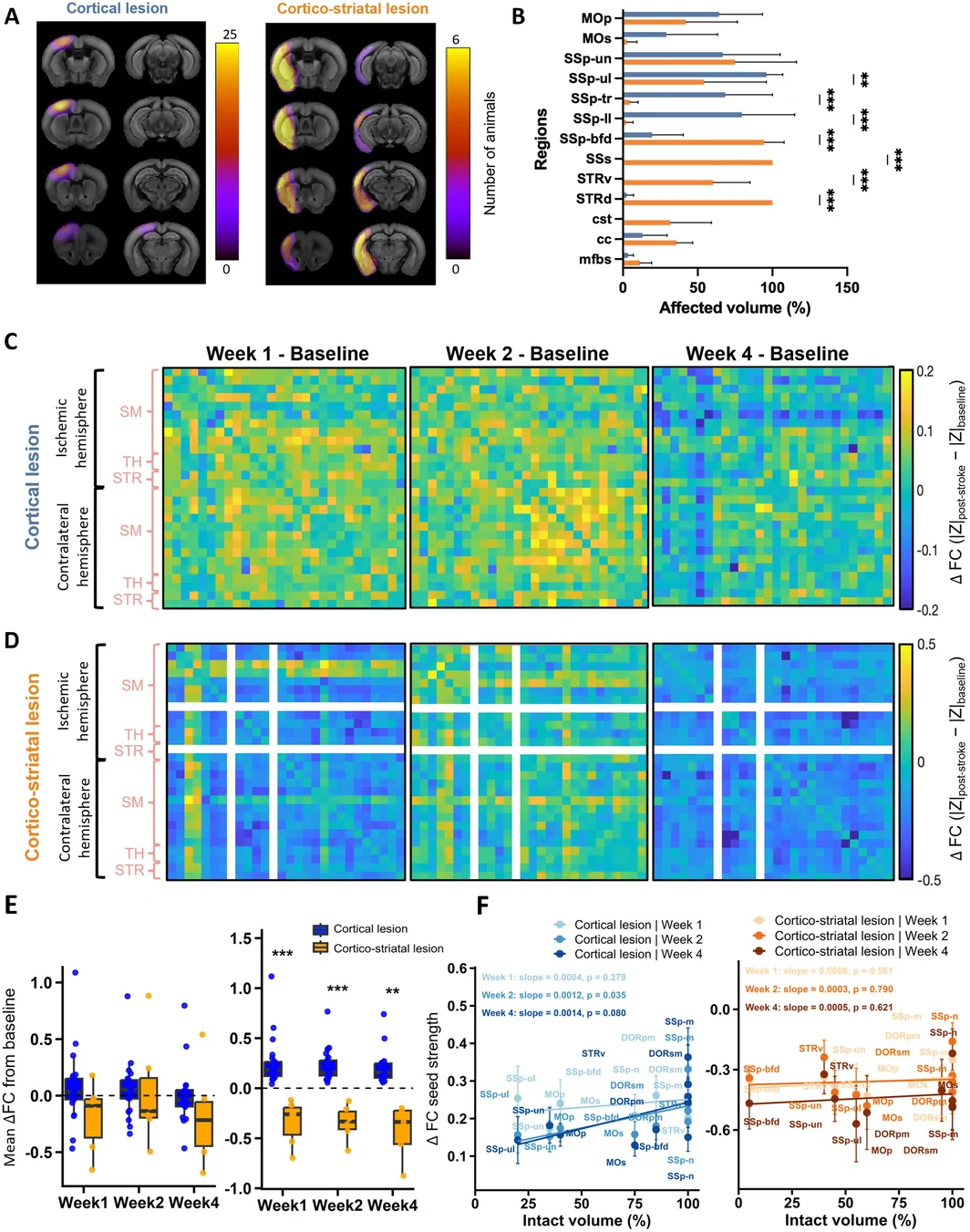
Lesion topography is associated with divergent baseline‐referenced FC changes in cortical vs. cortico‐striatal stroke. (A) Incidence maps were generated by averaging binary stroke masks in atlas space to compare lesion distribution between models. (B) Regional lesion volumes were quantified by overlaying masks with the brain atlas; group differences were tested with a mixed‐model post hoc test (*p < 0.05, **p < 0.01, ***p < 0.001). Data are mean ± SEM; cortical *n* = 25, cortico‐striatal *n* = 6. (C, D) Connectivity matrices show baseline‐referenced FC changes in the selected sensorimotor network at 1, 2, and 4 weeks post‐stroke. Matrices include all 28 bilateral ROIs, with network‐specific brackets indicating sensorimotor cortex, thalamus, and striatum; full region labels are provided in Figure S3. Color values show |*Z*|_post‐stroke_ − |*Z*|_baseline_, with yellow indicating increased and blue decreased absolute FC strength. In the cortico‐striatal lesion matrices in panel (D), white rows and columns indicate two regions that were 100% affected by the stroke lesion and were therefore completely excluded from FC calculations after removal of the lesion mask from the brain data. (E) Boxplots show mean baseline‐referenced ΔFC between cortical and cortico‐striatal lesion models. The left plot includes all connections among the 28 bilateral sensorimotor‐network ROIs, showing an overall tendency toward increased ΔFC after cortical lesion and decreased ΔFC after cortico‐striatal lesion, although group differences were not significant. The right plot shows mean ΔFC after restricting the analysis to 63 of 378 unique undirected connections that showed a consistent positive change from baseline in cortical lesions and a consistent negative change from baseline in cortico‐striatal lesions across all post‐stroke time points. This filtering reduced connection‐level variability and revealed significant differences between lesion models at each time point. Groups were compared using Welch's *t*‐tests (**p < 0.01, ***p < 0.001). (F) Scatter plots show the relationship between intact regional volume and ΔFC seed strength calculated from the filtered connections shown in panel (E). Intact regional volume was derived from the Week 1 lesion mask and defined as the percentage of each region not affected by the lesion, calculated as 100 − affected regional volume (%). ΔFC seed strength was calculated separately for cortical and cortico‐striatal lesions by averaging the baseline‐referenced ΔFC of the selected connections associated with each seed region. Points indicate individual seed regions and lines show linear fits for each post‐stroke time point. In cortical lesions, intact regional volume was significantly associated with ΔFC seed strength at Week 2 (slope = 0.0012, *p* = 0.035), with a positive trend at Week 4 (slope = 0.0014, *p* = 0.080); no significant associations were observed in cortico‐striatal lesions.

For each of the 98 atlas‐defined regions, a representative time series was obtained by averaging the voxel‐wise fMRI signal within the region. Pairwise Pearson correlations between all regional time series were calculated to generate FC matrices and transformed to Fisher *z*‐scores for statistical comparison and averaging. Seed strength was defined as the mean FC of a region with all other regions within a given matrix compartment, allowing assessment of intra‐ and interhemispheric connectivity over time (Figure 1). To minimize distortion from stroke‐related edema, lesion masks were generated from Week 1 T2‐weighted images, when lesion borders were most clearly visible, and were registered to the T2‐weighted images at subsequent time points before alignment to the corresponding rs‐fMRI/EPI scans (Figure S1). Lesioned voxels were longitudinally masked from regional time‐series extraction and FC computation while retained as zero‐valued spatial placeholders in the connectivity matrices; details are provided in the Appendix S1. For atlas‐based ROI definition we used the AIDAmri pipeline as described previously [20]. Briefly, from the Allen Mouse Brain Reference Atlas available from mouse.brain‐map.org (CCF v3, 2017), we generated a modified parental parcellation in which small subregions, such as individual cortical layers, were merged into their corresponding parent anatomical regions to reduce partial‐volume effects in EPI‐based rs‐fMRI data. This modified parcellation contained 49 regions per hemisphere, resulting in 98 bilateral ROIs. From this atlas, we selected stroke‐relevant regions within the sensorimotor network, including the primary somatosensory cortex (SSp: barrel field, SSp‐bfd; lower limb, SSp‐ll; mouth, SSp‐m; nose, SSp‐n; trunk, SSp‐tr; upper limb, SSp‐ul; unidentified area, SSp‐un; supplemental somatosensory area, SSs), primary and secondary motor cortex (MOp, MOs), thalamus (TH: postero‐medial, DORpm; somatosensory‐medial, DORsm), and striatum (dorsal, STRd; ventral, STRv). These 14 anatomical regions were analyzed separately in the ipsilesional and contralesional hemispheres, resulting in 28 bilateral ROIs for the targeted FC analyses. A complete list of included regions is provided in Table S1. For Figure 2E,F, baseline‐referenced ΔFC was calculated among the 28 bilateral ROIs of the targeted sensorimotor network, resulting in 378 unique undirected connections. Mean ΔFC was first evaluated across all 378 connections and then after filtering for connections with consistent opposite trajectories between models, defined as positive ΔFC in cortical stroke and negative ΔFC in cortico‐striatal stroke across all post‐stroke time points. This yielded 63 unique undirected connections. For Figure 2F, ΔFC seed strength was calculated from these filtered connections by averaging the baseline‐referenced ΔFC of connections associated with each seed region and relating it to intact regional volume, defined as 100 − affected regional volume (%) from the Week 1 lesion mask.

To characterize the temporal dynamics of individual connections, we focused on selected seed subnetworks centered on SSp‐ll, SSp‐ul, and MOp, which are key sensorimotor regions implicated in post‐stroke behavioral deficits. For each seed subnetwork, temporal FC patterns were classified as increased, decreased, or mixed according to the predominant direction of change across the contributing connections (Figure 4; Table 2).

**TABLE 2:** Summary of FC changes relative to baseline in key sensory‐motor regions.

		Cortical lesion			Cortico‐Striatal lesion		
		SSp‐ll	SSp‐ul	MOP	SSp‐ll	SSp‐ul	MOP
Ischemic hemisphere	Week1	↑	↑	↑	↓	↓	↓
	Week2	↑	↑	↑	↑	− −	− −
	Week4	− −	↓	↑	↓	↓	↓
Contralateral hemisphere	Week1	↑	↑	↑	↓	↓	↓
	Week2	↑	↑	↑	↑	↑	↓
	Week4	↑	− −	− −	↓	↓	↓
Ischemic → Contralateral	Week1	↑	− −	↑	↓	↓	↓
	Week2	− −	− −	↑	↑	↓	↓
	Week4	↓	↓	↑	↓	↓	↓
Contralateral → Ischemic	Week1	↑	↑	↑	↓	↓	↓
	Week2	↑	↑	↑	↓	− −	↓
	Week4	↑	− −	− −	↓	↓	↓
Cortical Homotopic	Week1	↓	↓	↑	↓	↓	↓
	Week2	↓	↓	↑	↓	↓	↓
	Week4	↓	↓	↑	↓	↓	↓

*Note:* Baseline‐referenced FC changes for SSp‐ll, SSp‐ul, and MOp at weeks 1, 2, and 4 after cortical and cortico‐striatal stroke. Predominant increase (red), decrease (blue), mixed (no clear dominant direction). ↑: Increase, ↓: Decrease, − −: mixed change.

### 2.3 Statistical Analyses

Longitudinal seed‐strength trajectories were analyzed using linear mixed‐effects models in R, with mouse as a random effect and day, region, and group as fixed factors. Seed strength was derived from Fisher‐z–transformed seed‐based FC matrices, and additional baseline‐referenced, lesion‐related, and between‐group difference‐in‐differences analyses were performed using estimated marginal means with multiple‐comparison correction. Full details on data preprocessing, model specification, post hoc testing, and overlap‐edge analyses are provided in the Appendix S1.

## 3 Results

### 3.1 Functional Connectivity in Two Different Stroke Models

At Week 1 T2w MRI showed a clear difference in lesion volume (*p* < 0.001) and anatomical distribution between the cortical and cortico‐striatal stroke (Figure 2A). Cortical strokes were smaller (1.41% ± 0.92% of total brain volume) compared to the cortico‐striatal stroke (11.53% ± 2.80%). Significant regional differences were observed (Figure 2B). Cortical strokes mainly affected the SSp‐ul (*p* < 0.01) and SSp‐ll (*p* < 0.001), whereas cortico‐striatal strokes more strongly affected SSp‐bfd, STRd, and STRv (*p* < 0.001). No significant differences were found in motor cortex or white matter damage, with the corticospinal tract, corpus callosum, and medial forebrain bundle only minimally affected in both models.

FC data were obtained from a modified Allen CCF 2017 atlas containing 49 regions per hemisphere, resulting in 98 bilateral ROIs and 4753 unique undirected ROI‐to‐ROI connections. Because this whole‐brain FC matrix contains a large number of connections across multiple longitudinal time points, subsequent analyses focused on selected sensorimotor, thalamic, and striatal regions implicated in post‐stroke recovery. FC in the two datasets was not different at baseline (pre‐stroke). There was a positive significant correlation between the normalized Day 0 FC profiles of the two groups. The normalized connection profiles were correlated using Pearson correlation (*r* = 0.653, *p* = 2.61 × 10^−47^; 95% CI = 0.591–0.707) and Spearman correlation (ρ = 0.516, *p* = 3.72 × 10^−27^). A mouse‐level bootstrap analysis further supported this finding, with a bootstrap Pearson median of *r* = 0.611 and 95% CI of 0.476–0.713 (Figure S2). *Cortical lesion group*: In both hemispheres, around 90% of the connections showed an increase during the first week (Figure 2C), which became even more pronounced by the second week in the contralateral intra‐hemispheric connections. However, by Week 4, FC had declined, most notably within the ischemic hemisphere, where approximately 50% of the connections showed a reduction. In contrast, the contralateral hemisphere maintained elevated FC levels compared to baseline. *Cortico‐striatal lesion group*: At 1 week post‐stroke, more than 90% of regions in both hemispheres showed reduced FC relative to baseline, with the exception of FC increase in the SSp‐n and SSp‐m. By the second week, both hemispheres displayed a slight FC increase, followed by a global decline again at Week 4 compared to the baseline (Figure 2D).

Using all 378 unique undirected connections among the 28 bilateral sensorimotor‐network regions, cortical lesions showed an overall tendency toward increased baseline‐referenced ΔFC, whereas cortico‐striatal lesions showed a tendency toward decreased ΔFC; however, these group differences were not significant (Figure 2E, left). To reduce connection‐level variability and focus on the most consistent model‐specific changes, we next restricted the analysis to 63 of 378 unique undirected connections that showed a consistent positive change from baseline in cortical lesions and a consistent negative change from baseline in cortico‐striatal lesions across all post‐stroke time points. This filtered connection set clearly separated the two stroke models at each post‐stroke time point (Figure 2E, right). To assess whether these divergent FC changes were related to regional lesion involvement, we calculated ΔFC seed strength from the filtered connections and related it to intact regional volume, defined as 100 − affected regional volume (%) (Figure 2F). Intact regional volume was derived from the Week 1 lesion mask, which was used for all post‐stroke time points because lesion borders were most reliably visible at Week 1 (Figure S1). In cortical lesions, ΔFC seed strength showed positive slopes across post‐stroke time points, with a significant association at Week 2 (slope = 0.0012, *p* = 0.035) and a positive trend at Week 4 (slope = 0.0014, *p* = 0.080). In contrast, cortico‐striatal lesions showed persistently negative ΔFC seed strength with little dependence on intact regional volume, and no significant regression slopes (Figure 2F).

In summary, the two stroke models showed distinct FC trajectories. Cortical lesions produced increases relative to baseline, especially contralesionally, whereas cortico‐striatal lesions showed early widespread decreases, partial rebound at Week 2, and renewed decline by Week 4. A subset of 63 of 378 unique undirected connections with consistent opposite changes between models separated the groups across post‐stroke time points, and the relationship between Week 1 lesion‐mask‐derived intact regional volume and ΔFC seed strength differed between lesion models.

### 3.2 Seed Strength

**FIGURE 3:**
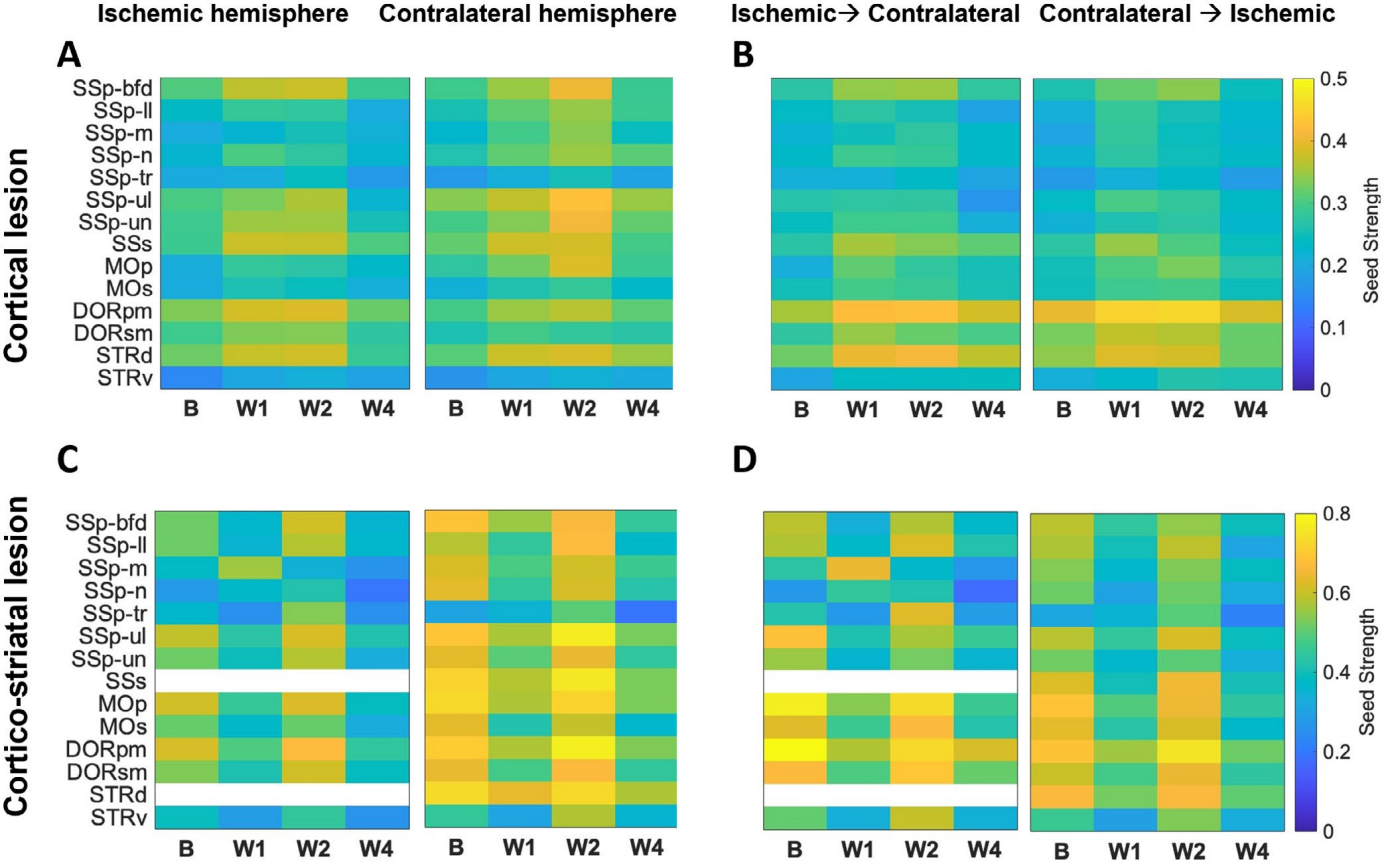
Two‐dimensional visualization of unilateral and bilateral seed strength changes in the ischemic and contralateral hemispheres over time. Seed strength changes were analyzed separately for the cortical and cortico‐striatal stroke models in both hemispheres (panels A and C), as well as for the connections from the ischemic to contralateral hemisphere and vice versa (panels B and D). Regions that were entirely affected by the stroke (~100% infarcted) are shown in white and were excluded from the analysis because no measurable seed strength changes could be observed.

Given the clear FC differences between the two stroke models, we next assessed longitudinal seed strength, defined as the total FC of each region with the rest of the brain (Figure 3). No within‐group baseline comparisons remained significant after Tukey correction. At the uncorrected level, four contrasts reached *p* < 0.05, all in the cortical group: contralateral Week 2 (MOp, SSp‐m, SSp‐un) and ischemic‐to‐contralateral Week 1 (MOp). For MOp, the 95% CIs were [−0.239, −0.006] at contralateral Week 2 and [−0.218, −0.006] at ischemic‐to‐contralateral Week 1, but these effects did not survive correction (Table S2).

In the cortical lesion group, seed strength increased globally at Weeks 1–2; particularly in the SSp‐bfd, SSp‐ul, SSp‐un, SSs, as well as DORpm and STRd. By Week 4, seed strength values returned to baseline levels (Figure 3A). The seed strength between hemispheres showed a bi‐directional increase at weeks 1 and 2; particularly in the DORpm and STRd. By week 4, seed strength values had returned to baseline levels (Figure 3B).

In the cortico‐striatal group, seed strength in the ischemic hemisphere decreased at Week 1, except in SSp‐n and SSp‐m, which increased relative to baseline. At Week 2, seed strength increased in most regions except SSp‐m, while by Week 4 a global decrease was observed. The contralateral hemisphere showed a similar pattern but with further increases at Week 2 (Figure 3C). Interhemispheric seed strength with ischemic‐hemisphere seeds decreased in most regions at Week 1, except SSp‐m and SSp‐n, which increased relative to baseline, mirroring intra‐hemispheric connectivity in the ischemic hemisphere. At Week two, most regions increased toward baseline, while SSp‐m and SSp‐n decreased relative to Week 1. By Week 4, all regions showed reduced interhemispheric seed strength compared to baseline. In the contralateral hemisphere, interhemispheric seed strength followed the same pattern as contralateral intra‐hemispheric connectivity: a decrease at Week 1, recovery at Week 2, and another reduction at Week 4 (Figure 3D).

For the between‐group comparison of trajectories, baseline‐referenced difference‐in‐differences (DiD) contrasts (Δcortical stroke − Δcortico‐striatal stroke) showed no significant effects after applying single‐step multivariate *t* (mvt) correction to control the family‐wise error rate across all DiD tests within each hemisphere. At the uncorrected level, 25 region‐by‐day DiD contrasts reached *p* < 0.05, primarily at Week 1. For MOp, nominal DiD effects were observed across multiple hemispheres and time points with 95% confidence intervals for the estimated contrast of contralateral‐to‐ischemic at Week 1 [0.032, 0.536] and Week 4 [0.004, 0.511], ischemic at Week 1 [0.051, 0.510] and Week 4 [0.035, 0.498], and ischemic‐to‐contralateral at Week 1 [0.081, 0.608] and Week 4 [0.095, 0.625] (Table S3). Together, the seed‐strength analysis indicates that functional network changes were region‐ and time‐specific, occurring mainly in the ischemic hemisphere after cortical strokes and in the contralateral hemisphere after cortico‐striatal strokes. Effect sizes were modest and variability across animals was high, reducing estimated precision and preventing adjusted *p*‐values from reaching significance.

### 3.3 Temporal Dynamics of Individual Connections in Somatosensory Regions

**FIGURE 4:**
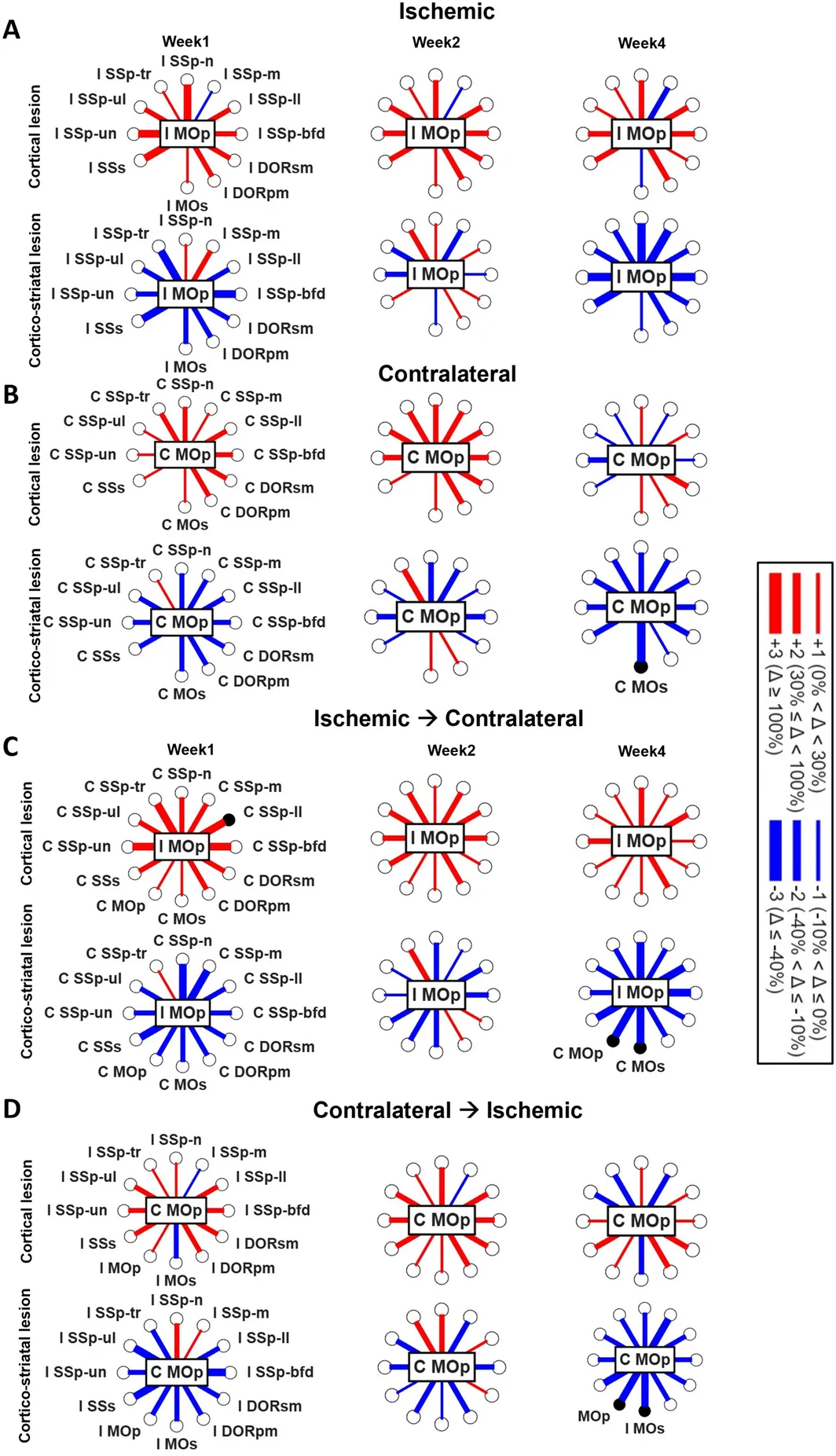
Connectivity changes in the MOp network. Changes in FC compared to baseline within the ischemic (A), contralateral hemisphere (B), ischemic to contralateral (C) and contralateral to ischemic connections (D). Decrease (blue) and increase (red) compared to baseline; line weight encodes change in magnitude; black‐filled points label a statistically significant change from baseline for that target region at that time point (linear mixed‐effects model with post hoc contrasts of Day within Region, adjusted for multiple comparisons; *p* < 0.05).

We next examined the individual connections underlying seed‐strength changes in SSp‐ul, SSp‐ll, and MOp. In the cortical group, MOp‐centered subnetworks were predominantly increased relative to baseline, with > 80% of connections enhanced in the ischemic hemisphere and 100% in the contralateral hemisphere at Weeks 1–2, followed by a more mixed pattern at Week 4 (Figure 4A–D). Interhemispheric connections were also consistently increased, especially between MOp and SSp‐ll. In contrast, the cortico‐striatal group showed marked disruption, with about 80%–92% of connections decreasing at Week 1, a partial or mixed pattern at Week 2, and nearly complete or complete decreases by Week 4. The strongest reductions involved motor–motor and motor–somatosensory connections, particularly between MOp, MOs, and SSp‐ll (Figure 4A–D).

Detailed connection‐level analyses for SSp‐ll and SSp‐ul are provided in Figures S4, S5 and show seed‐specific deviations from the MOp pattern, including more sustained contralateral increases after cortical stroke and a more pronounced transient Week‐2 rebound after cortico‐striatal stroke before decline by Week 4.

### 3.4 Patterns of Increased and Decreased FC

To summarize the pattern of FC changes across the two stroke models, we assessed the trends within the sub‐networks that contributed to the seed strength, focusing on whether they showed a general increase or decrease in strength. Table 2 provides a simplified seed‐wise summary of the detailed connection‐level FC changes shown in Figure 4, classifying each selected seed region as showing a predominantly increased, decreased, or mixed pattern relative to baseline. Longitudinal changes in FC across selected seed regions (SSp‐ll, SSp‐ul, and MOp) following cortical and cortico‐striatal stroke revealed distinct patterns of reorganization. In cortical stroke, the majority of the assessed connections increased post‐stroke but decreased in the cortico‐striatal stroke—both within (intra‐hemispheric) and between (inter‐hemispheric) hemispheres—highlighting a model‐specific directionality in FC reorganization. In examining homotopic cortical connections, SSp‐ll and SSp‐ul demonstrated similar patterns of change across both stroke models, generally trending toward reduced FC. In contrast, the MOp seed region exhibited an opposing pattern: its homotopic FC increased in the cortical stroke model but decreased in the cortico‐striatal stroke model. This divergence suggests that the MOp network may be particularly sensitive to the involvement of subcortical structures in stroke pathology (Table 2).

## 4 Discussion

We combined longitudinal rs‐fMRI with atlas‐based analysis in two stroke models. Cortico‐striatal strokes caused early global hypoconnectivity, transient recovery, then widespread reduction, whereas cortical strokes induced early hyperconnectivity partially reversed by Week 4. Seed‐strength mirrored these trends, though between‐model differences were subtle. Connection‐wise analysis identified a small set of interhemispheric links, especially MOp–MOp and SSp‐ll–SSp‐ll, that clearly distinguished the two stroke models, with homotopic coupling increasing in MOp after cortical stroke but decreasing after cortico‐striatal stroke.

### 4.1 FC Changes in the Cortical Stroke Model

In the cortical stroke model, around 90% of connections increased during Weeks 1 and 2 post‐stroke, followed by a decrease in approximately half of the connections in the ischemic hemisphere by Week 4. This early FC increase is in line with previous findings in photothrombotic stroke and cortical distal MCAO in mice at 2 weeks [14]. In contrast, functional optical intrinsic signal imaging (fcOIS), which relies on hemodynamic signals, reported pronounced FC reductions at 2 weeks post‐stroke [22]. The discrepancy may reflect time‐dependent dynamics, given that the fcOIS study included only a single post‐stroke time point.

At the level of transhemispheric homotopic connections, which decreased over time, and intrahemispheric contralesional sensorimotor connections, which increased, our rs‐fMRI results are broadly consistent with rs‐fMRI and optical imaging reports, although the exact regions analyzed do not fully overlap across studies. Wide‐field calcium imaging reported reduced interhemispheric connectivity when the contralateral forelimb area was used as the seed region [23], supporting our observation that homotopic coupling weakens over time. In our analysis, FC in homotopic primary somatosensory regions, but not in the motor cortex, decreased from Week 1 to Week 4. This is in line with rs‐fMRI and calcium imaging studies reporting stronger FC reductions in somatosensory than in motor cortex [14, 24]. This pattern may relate to lesion topography in our photothrombotic model, where the infarct predominantly affected somatosensory cortex but largely spared motor cortex, suggesting that FC declines preferentially in regions closer to the lesion core. In contrast, parts of the somatosensory thalamocortical loop showed increased connectivity, consistent with an early compensatory response in both hemispheres that has been linked to functional recovery in an rs‐fMRI study in stroke patients [25].

### 4.2 FC Changes in the Cortico‐Striatal Stroke Model

In the cortico‐striatal stroke model there was an early FC decrease in more than 80% of connections, which was partially reversed at 2 weeks but later manifested at 4 weeks with all connections showing decreased FC. The seed‐strength trajectories mirrored these dynamics without any specific intrahemispheric pattern or directionality (ischemic to contralateral vs. contralateral to ischemic). Perilesional areas, specifically the somatosensory mouth and nose areas were the only two regions that showed an opposite pattern, i.e., FC increase compared to baseline at Weeks 1 and 2. The acute FC decline is in line with previous MCAO studies that focused on interhemispheric connectivity in rats using rs‐fMRI at 3–70 days post‐stroke [26] and on inter‐ and intrahemispheric connectivity in mice using fcOIS at 2 weeks post‐stroke [27]. When the striatum or basal ganglia are involved, stroke disrupts the cortico‐striatal and thalamo‐cortical loops that normally stabilize long‐range cortical integration [28]. This disruption compromises key hubs that sustain communication between cortex and thalamus [29], leading to early, widespread hypoconnectivity followed by a transient rebound and subsequent decline. Such patterns might reflect deeper and longer lasting diaschisis together with hub loss [30]. Cortical homotopic FC of the somatosensory and motor regions were uniformly reduced at all time points post‐stroke, whereas in a comparable cortico‐striatal stroke study in rats this decrease was only transient compared to baseline (up to 3 weeks) [26]. Lesions extending into the basal ganglia or interhemispheric fibers further promote maladaptive interhemispheric inhibition, typically persistent contralesional suppression of ipsilesional M1 activity, and hinder the renormalization of motor networks [3, 31].

### 4.3 Role of Lesion Size and Location for FC Changes

After a stroke, functional recovery depends not solely on the initial damage, but to an increasingly recognized extent also on lesion topology [32] and on how large‐scale networks rebalance in the first weeks [3]. Stroke lesions disrupt interhemispheric coupling and trigger diaschisis in connected regions [33]. Early hypoconnectivity has been linked to lesion size in both transient MCAO and photothrombotic stroke models [22, 26, 34]. Here, lesion topography shaped both the direction and robustness of FC change: A subset of 63 unique undirected sensorimotor connections showed consistent divergent trajectories between cortical and cortico‐striatal stroke across all post‐stroke weeks, indicating model‐specific network responses rather than transient fluctuations. In cortical stroke, when the remaining intact part of the affected regions was larger, FC showed stronger increase compared to baseline. This may reflect functional remapping, i.e., the topographical shift of functional activation emerging between 2 and 4 weeks post‐stroke [35] or compensatory upregulation of spared tissue through potentiation of pre‐existing circuits [24]. In the cortico‐striatal group, pre‐selected for a decrease in FC, there was no significant relationship with the size of the affected regions. Thus, FC reductions may be driven less by the exact amount of damage within an individual brain region and more by the stroke‐induced breakdown of distributed sensorimotor networks with no clear signs of local cortical remapping [26, 27].

In the full dataset, robust motor‐cortex changes were limited and similar in both models, including lower FC at Week 4 and a transient MOp–SSp‐ll increase at Week 1. This aligns with experimental and clinical evidence that subcortical involvement is associated with broader network disruption and poorer functional outcomes, whereas purely cortical infarcts may show more variable FC changes and greater recovery potential [36, 37].

In humans, larger lesions involving subcortical nuclei are associated with greater disruption of corticocortical FC and poorer behavioral outcomes, whereas purely cortical infarcts show more variable connectivity changes and recovery potential [3, 38]. Indeed, prediction of recovery is therefore strongly influenced by lesion topography and structural connectivity [31, 33]. In our data, the relationship between lesion involvement and FC change was model‐ and time‐dependent rather than uniform across both stroke models. In cortical stroke, greater intact regional volume was associated with stronger ΔFC seed strength at week 2, with a similar positive trend at week 4. This suggests that FC increases may partly depend on preserved regional tissue and could reflect compensatory network upregulation [39]. In contrast, cortico‐striatal stroke showed persistently negative ΔFC seed strength with little dependence on intact regional volume, indicating that sustained FC reductions in this model were not explained by local regional involvement alone, but may instead reflect broader sensorimotor‐network disruption after lesions involving both cortical and subcortical tissue [7]. Similar phenomena, in which the spared cortex supports adaptive upregulation of networks, have been observed in both experimental and human fMRI studies [25, 40], with the strength of upregulated FC often predicting better recovery.

The divergent FC trajectories, however, cannot only be attributed to lesion size and location but must also be interpreted in light of the differences in the underlying pathology between both models. Cortical photothrombotic lesions lead to focal endothelial damage, rapidly developing ischemic cell death but typically lack a penumbra and reperfusion, whereas MCAO produces early striatal followed by delayed cortical infarction, penumbral tissue and potential reperfusion injury [12]. Both models show blood–brain barrier disruption for several weeks, while more focal in photothrombosis, in both it can spread to other regions and has been linked to secondary degeneration (SND) processes [41]. SND has been shown to evolve over days to weeks after cortical stroke and to affect remote cortical and subcortical regions, particularly the ipsilesional thalamus and thalamocortical pathways [42, 43]. In photothrombotic cortical stroke, secondary degeneration has been reported mainly in dorsal sensorimotor thalamic regions and related thalamocortical tracts accompanied by neuroinflammation [18, 44]. Other brain regions susceptible to degeneration have been linked to transiently increased FC in human studies, potentially reflecting compensatory or maladaptive network reorganization [45, 46]. Although a direct correlation between SND and FC alterations has not yet been established, the increased FC observed in our cortical PT model, particularly in interhemispheric DORpm connections, could similarly reflect transient network reorganization. In contrast, cortico‐striatal stroke may disrupt broader long‐range cortico‐subcortical projections, thereby limiting compensatory reorganization and contributing to sustained FC reductions at later stages [26].

Importantly, the present study leverages an edge‐centric approach to directly link lesion involvement to both the *direction* and *statistical strength* of FC changes, extending prior work that largely focused on overall FC magnitude or behavioral correlations without systematically evaluating lesion volumetrics [47]. Our findings also resonate with emerging evidence that lesion size alone does not fully account for recovery after stroke. Humans and rodent studies have shown that the lesion location and lesion‐affected networks relate to the capacity for reorganization [48, 49, 50, 51].

In summary, our data demonstrate that lesion topography not only determines the *direction* of FC change but also modulates the *statistical strength* of reorganization. These results support a circuit‐level framework for understanding post‐stroke network dynamics, in which the anatomical context of damage, rather than infarct volume alone, shapes the functional trajectories of recovery.

### 4.4 Comparison With Structural Connectivity Data

In a previous analysis [15], we found that large cortico‐striatal lesions led to increased structural connectivity (SC) in sensorimotor regions, whereas small cortical lesions induced asymmetric connectivity changes: an increase extending globally from the ischemic hemisphere and a decrease expanding globally from the healthy hemisphere. One week after the transient FC increase at Week 1, SC in primary somatosensory, thalamic, corpus callosum, and corticospinal tract (cst) increased. The increase in FC before SC likely reflects an immediate functional network response that precedes slower, diffusion MRI‐detectable axonal remodeling, as shown in a transgenic mouse model of cerebral amyloidosis [52]. FC increases despite reduced corpus callosum SC further support a possible FC–SC dissociation, as also reported in humans with callosal agenesis [53]. After cortico‐striatal MCAO, contralesional SC of sensorimotor and thalamic connections increased, whereas ipsilesional striatal and white‐matter degeneration emerged [15]. Here, the FC pattern, i.e., global FC decrease at Week 1, transient rebound at Week 2, especially in the contralateral hemisphere, and again FC decrease at Week 4, is compatible with disrupted corticostriatal and thalamo‐cortical loops followed by a temporary shift toward contralesional networks, which can be interpreted as post‐stroke adaptive reorganization toward [26, 54].

### 4.5 Methodological Limitations

A limitation of this study is that the cortical PT and cortico‐striatal MCAO cohorts were obtained from previously established longitudinal MRI studies and were not originally designed as a fully balanced head‐to‐head comparison. Although animal handling, anesthesia protocols, atlas‐based preprocessing, and FC analysis strategies were harmonized across cohorts, other experimental factors could not be fully excluded as potential confounders. The groups differed in sample size, mouse strain, stroke model, and lesion location/extent. In particular, the PT cohort was acquired in C57BL/6J mice, whereas the MCAO cohort was acquired in NMRI‐nu mice; therefore, strain‐related effects cannot be fully separated from model‐related differences. To our knowledge, no prior direct rs‐fMRI comparison between C57BL/6J and NMRI‐nu mice exists. However, strain‐related differences in mouse brain function and connectivity have been reported in other comparative imaging studies, including differences in neuronal activity and resting‐state network organization relevant to sensorimotor networks [5, 55]. To address potential baseline differences between cohorts, we performed an additional Day 0 FC‐pattern analysis. This analysis showed that the baselines of the groups were broadly similar between cohorts, although absolute baseline FC magnitude differed. Therefore, between‐model differences were interpreted descriptively and based on within‐subject baseline‐referenced FC changes rather than direct comparisons of raw FC magnitude.

A further limitation of this study is the unequal sample size between the cortical stroke and cortico‐striatal stroke groups. In particular, the smaller number of animals in the cortico‐striatal MCAO group reduced statistical power and may have limited the detection of subtle FC changes. Therefore, differences in the number of significant connections or seed‐strength alterations between models should be interpreted cautiously, as they may partly reflect differences in statistical sensitivity. For this reason, we avoided direct inferential statistical comparisons between stroke models and focused primarily on longitudinal within‐model changes relative to each model's pre‐stroke baseline. Furthermore, some statistical comparisons may not have reached significance because of stringent correction for multiple comparisons. For example, seed‐strength analysis required correction across a large number of pairwise comparisons, which may have reduced sensitivity to small or spatially heterogeneous effects. Another limitation is that behavioral assessments were not uniformly available and were not harmonized across the two stroke‐model datasets. Although previously published source studies reported sensorimotor impairment after both photothrombotic cortical stroke and 30‐min MCAO, the behavioral tests, time points, and included animals differed between datasets. Therefore, the present study was not designed to directly compare behavioral severity or recovery duration between models, nor to test FC–behavior relationships across cohorts. The observed model differences should therefore be interpreted as differences in longitudinal FC trajectories rather than direct evidence of poorer or longer lasting behavioral impairment in one model. Future studies should use balanced group sizes, harmonized mouse strains, matched MRI protocols, and standardized behavioral testing to improve detection of longitudinal trajectory effects and directly assess links between lesion topography, FC/SC reorganization, and behavioral recovery across stroke models.

## 5 Conclusion

With this first direct comparison of longitudinal FC changes in cortical and cortico‐striatal strokes in mice, we provide a framework for interventional studies targeting the rebalance of FC and network stabilization in relation to functional recovery after stroke. The in‐depth atlas‐based characterization provides an opportunity to design region‐ and connection‐specific neuromodulation and pharmacological therapies building on the spontaneous FC dynamics during the acute, sub‐acute, and chronic weeks after stroke.

## Author Contributions

**Fatemeh S. N. Mahani:** methodology, software, formal analysis, data curation, writing, visualization. **Michael Diedenhofen:** methodology, software, data curation, formal analysis. **Claudia Green:** methodology, investigation, data curation. **Dirk Wiedermann:** methodology, software, resources. **Gereon R. Fink:** writing, supervision, and funding acquisition. **Mathias Hoehn:** conceptualization, methodology, formal analysis, writing, visualization, supervision. **Markus Aswendt:** conceptualization, methodology, resources, data curation, writing, visualization, supervision.

## Ethics Statement

The animal experiments were performed in accordance with the ARRIVE [56] and IMPROVE [57] guidelines. The experiments were conducted under the German Animal Welfare Act and approved by the Landesamt für Natur, Umwelt und Verbraucherschutz North Rhine‐Westphalia (animal permission IDs 84‐02.04.2014.A305 from 2015‐01‐26 and 84‐02.04.2016.A461 from 2017‐07‐06, Max Planck Institute for Metabolism Research, and 81‐02.04.2019.A309 from 2019‐11‐25, University Hospital Cologne).

## Conflicts of Interest

The authors declare no conflicts of interest.

## Supporting information


**Figure S1:** Representative lesion‐mask registration and lesion‐exclusion procedure in functional space. Representative examples from the cortical PT and cortico‐striatal MCAO groups showing how lesion masks were generated and applied during preprocessing. For each model, the upper row shows a Week 1 T2‐weighted anatomical image without and with the manually delineated stroke mask overlaid in red. The lower row shows the corresponding rs‐fMRI/EPI image, the atlas region after subtraction of the registered lesion mask, and the atlas overlay in functional space. Because the lesion is not reliably visible on EPI/rs‐fMRI images, lesion masks were delineated on T2‐weighted images and then registered to the corresponding functional images. Lesion‐affected voxels were excluded from regional time‐series extraction and FC computation while preserving the spatial representation of affected regions for visualization.
**Figure S2:** Day 0 FC‐pattern similarity between PT and MCAO cohorts. Day 0 FC values were collapsed into unique undirected connections and normalized within each mouse to reduce the influence of global FC magnitude differences. The group‐average normalized connection profiles were positively correlated between PT and MCAO, indicating broadly similar baseline FC organization despite differences in mouse strain, stroke model, and absolute baseline FC magnitude.
**Figure S3:** Fully labeled FC matrices for the sensorimotor network. Adjacency matrices showing FC changes from baseline to Week 1 after stroke in the cortical (A) and cortico‐striatal lesion model (B). The matrices include all 28 bilateral ROIs used in the FC analysis, corresponding to 14 anatomical regions in each hemisphere (I—ischemic and C—contralateral). Regions are grouped by hemisphere and network, including the sensorimotor cortex, thalamus, and striatum. Color values indicate the difference in absolute Fisher z‐transformed functional connectivity between Week 1 and baseline, Positive values indicate increased absolute FC strength relative to baseline, whereas negative values indicate decreased absolute FC strength relative to baseline.
**Figure S4:** Connectivity changes in the SSp‐ll network. Relative changes in FC compared to baseline within the ischemic (A), contralateral hemisphere (B), ischemic to contralateral (C), and contralateral to ischemic connections (D). The color blue indicates a weakening of connections following stroke, while red represents a strengthening of connections relative to baseline; line weight encodes the magnitude of change (legend). Black‐filled points mark connections showing a statistically significant change from baseline for that target region at that time point (linear mixed‐effects model with post hoc contrasts of Day within Region, adjusted for multiple comparisons; *p* < 0.05).
**Figure S5:** Connectivity changes in the SSp‐ul network. Relative changes in FC compared to baseline within the ischemic (A) and contralateral hemisphere (B) as well as for the ischemic to contralateral (C) and contralateral to ischemic connections (D). The color blue indicates a weakening of connections following stroke, while red represents a strengthening of connections compared to baseline; line weight encodes change magnitude (legend). Black‐filled points mark connections showing a statistically significant change from baseline for that target region at that time point (linear mixed‐effects model with post hoc contrasts of Day within Region, adjusted for multiple comparisons; *p* < 0.05).
**Table S1:** Selected regions used for targeted FC analysis.
**Table S2:** Within‐group longitudinal statistics for baseline‐referenced FC. Region‐ and hemisphere‐resolved results from linear mixed‐effects (LME) models assessing changes over time within each stroke group. For each group and region (ipsi−/contralesional hemisphere), model‐based contrasts compare each post‐stroke time point to the pre‐stroke baseline and report the corresponding effect estimate, with 95% confidence interval (CI) and *p*‐value. *p*‐values shown are uncorrected (nominal *p* < 0.05); after multiple‐comparisons correction (post hoc adjustment across regions/contrasts), none of the effects remained significant (*p*_adj_ ≥ 0.05).
**Table S3:** Between‐group statistics for baseline‐referenced functional connectivity changes. Region and hemisphere‐resolved between‐group comparisons of baseline‐referenced FC change (ΔFC = FC_post_−FC_baseline_) at each post‐stroke time point. ΔFC was analyzed using LME‐based contrasts to test group differences per region and hemisphere at Weeks 1, 2, and 4, and results are reported as effect estimate, 95% CI, and *p*‐value. *p*‐values shown are uncorrected (nominal *p* < 0.05); after multiple‐comparisons correction, no comparisons remained significant (*p*_adj_ ≥ 0.05).

## Data Availability

The dataset including all raw and processed MRI data, custom scripts, and code is available at DOI: 10.12751/g‐node.pmvtz1 (CC BY‐NC‐SA 4.0 license) according to the scheme explained in Ref. [58].
