# Supplementary material for: Dynamic Functional Connectivity Changes in Cortical and Cortico‐Striatal Strokes in Mice
Source: CNS Neurosci Ther. 2026 Aug 27;32(8):e71114. doi: 10.1002/cns.71114 (PMC13522393; doi:10.1002/cns.71114)
Supplement: Supplementary file 1 [file CNS-32-e71114-s001.docx]

# Supplementary Material

## MRI processing

Preprocessing using AIDAmri [1] included Bruker-to-NIfTI conversion with brkraw, skull stripping with FSL BET, correction of bias field inhomogeneities using MICO [2], slice-timing and motion correction, regression of respiratory-related fluctuations, band-pass filtering (0.01–0.1 Hz), and spatial smoothing (Gaussian kernel, sigma 0.1 mm). T2-weighted images were aligned to atlas space using affine and non-linear transformations, and fMRI data were coregistered to the corresponding anatomical scans for extraction of atlas-based regional signals from 98 regions. Stroke lesions were identified as hyperintense regions on T2-weighted MRI and segmented semi-automatically in ITK-SNAP using the active contour tool [3]. Lesion masks were registered to atlas space to generate lesion incidence maps and quantify regional lesion involvement. Stroke-induced brain edema, caused by fluid accumulation, can significantly distort brain anatomy on MRI and interfere with accurate FC analysis. To mitigate these effects, we implemented a lesion-masking approach that excludes stroke-affected voxels from contributing to regional time series while preserving their location within the data structure. Lesion masks generated at week 1 were registered to T2-weighted images at all subsequent time points and then aligned to the corresponding fMRI scans. The transformed lesion areas were set to zero in the time-series maps at each time point, effectively nullifying their contribution to signal analysis while preserving their spatial positions for downstream visualization. This adjustment was applied throughout the processing pipeline. Anatomical segmentations and rs-fMRI data were modified to exclude lesioned voxels from regional averaging while retaining zero-value regions to represent stroke-affected areas in the connectivity matrices. Seed ROIs were redefined to omit lesion voxels during time-series extraction, ensuring only intact tissue signals were analyzed. Pairwise Pearson correlations were then computed between non-lesional regions, producing connectivity matrices in which stroke-affected regions appear as zero-valued entries, preserving the network’s spatial structure.

## Statistics

Longitudinal changes in seed strength were analyzed using linear mixed-effects models (LMMs) in R (v4.4.1). Models were fitted using lmer() from the **lmerTest** package, and post-hoc contrasts were performed using **emmeans**. Data handling and preprocessing were performed with tidyverse tools (**dplyr**, **readr**, **stringr**, **tibble**, **purrr**), and model fitting relied on **lme4**. Data preparation and outcome definition (seed-level subnetworks): Seed strength values were derived from seed-based FC matrices. For each input CSV file, connection-level Fisher-z–transformed FC values were read for each mouse and time point. Day labels were standardized to numeric post-stroke days; baseline measurements were coded as **Day 0**, and analyses focused on **Day 0, 7, 14, and 28**. For each **Hemisphere × Seed (Region) × Group × MouseID × Day**, we computed a mouse-level seed strength summary by averaging Fisher-z FC across all connections belonging to that seed. This produced one observation per mouse per time point (per seed and hemisphere), reducing connection-level data to a longitudinal seed-strength trajectory while retaining within-animal repeated measures. Model structure and fixed/random effects: All models included MouseID as a random intercept to account for repeated measurements within each animal and to accommodate inter-subject variability in baseline seed strength. Time was treated as a categorical factor (Day_f) with levels {0, 7, 14, 28}, allowing comparisons to be made between specific post-stroke time points and baseline without assuming linearity over time. The seed region was treated as a factor (Region) corresponding to the seed/ROI identity. Analyses were conducted separately for each hemisphere, yielding hemisphere-specific inferences for seed strength trajectories. For the within-group longitudinal analysis, a mixed-effects model was applied: within each hemisphere, longitudinal changes were modelled separately for each group (PT or MCAO) using:

**SeedStrength ∼ Day_f_ × Region + (1 ∣ MouseID),**

where **SeedStrength** is the mouse-level, seed strength for a given hemisphere/region/day; **Day_f_** represents measurement day (0, 7, 14, 28); **Region** represents the seed region; and the interaction **Day_f_ × Region** tests whether the time course differs between regions (i.e., whether temporal change depends on region). Post-hoc tests were based on estimated marginal means from the mixed model. Within each region, we compared all pairs of days and report both unadjusted p-values and Tukey-adjusted p-values to control the family-wise error rate for the within-region day comparisons.

To relate regional lesion involvement to baseline-referenced FC changes within each stroke model, we used the subset of sensorimotor edges that showed consistent, opposite directionality between groups across all post-stroke time points (the “overlap edge set”; defined in the Results). For each mouse, time point (Day 0, 7, 14, 28), and atlas region, we computed **ischemic-hemisphere seed strength** as the mean **raw FC** across all edges in the overlap edge set targeting that region, combining intra- and inter-hemispheric edges. For each animal and week, the mean ΔFC across the selected connections was calculated and compared between groups using two-sample t-tests (Welch’s correction).

Baseline-referenced changes in regional seed strength were then tested using the within-group LMM framework described above (random intercept for MouseID; Day × Region fixed effects; Day treated as a categorical factor). For each region, baseline contrasts (week 1 vs 0, week 2 vs 0, week 4 vs 0) were obtained from estimated marginal means and corrected for multiple comparisons using Tukey adjustment. To visualize how lesion burden related to the strength of baseline-related FC changes, regional lesion extent (%) was plotted against −log10(p_Tukey) from the corresponding baseline contrast, with larger values indicating stronger statistical evidence for deviation from baseline. Between-group analysis of trajectories using a mixed-effects model: To test whether the 2 stroke models showed different longitudinal trajectories, a combined model was fitted within each hemisphere:

**SeedStrength ∼ Day_f_ × Region × Group + (1 ∣ MouseID),**

where **Group** is a two-level factor (PT vs MCAO). This specification allows: overall differences between groups, time effects that vary by region, and critically, whether **group differences over time depend on region** (three-way interaction).
Baseline-referenced difference-in-differences (DiD) post-hoc contrasts: Rather than relying only on raw interaction coefficients, between-group inference was expressed as baseline-referenced **difference-in-differences (DiD)** contrasts for each region and post-stroke day d∈{7,14,28}:

**ΔΔSeedStrength(d) = (FC_cortical,d_ −FC_cortical,0_) − (FC_cortico-strital,d_−FC_cortico-striatal,0_)**

These DiD contrasts quantify whether the **change from baseline** differs between cortical and cortico-striatal stroke models at each post-stroke day, within each region. DiD contrasts were implemented by applying custom linear contrasts applied to the EMM grid from the fitted three-way model. For post hoc analysis, we tested all baseline-referenced difference-in-differences (DiD) contrasts across regions and post-stroke days within each hemisphere, reporting both unadjusted p-values and single-step multivariate t–adjusted p-values (mvt) to control the family-wise error rate across all DiD tests within that hemisphere.

## Temporal dynamics of individual connections in somatosensory regions

SSp-ll followed a somewhat different trajectory (**Fig. S4**). In cortical stroke, contralateral connectivity stayed uniformly elevated at every time point (100%) (**Fig. S4B**). On the ischemic side, SSp-ll showed strong increases at weeks 1–2 (>80%) but shifted to a mixed pattern by week 4; within this ipsilesional set, connectivity to MOp remained predominantly increased (**Fig. S4A**). Across hemispheres, ischemic-to-contralateral increased at week 1 (67%), became mixed at week 2, and then reversed toward a mainly decreased pattern by week 4 (83% decreased) (**Fig. S4C**). By contrast, contralateral-to-ischemic connectivity remained consistently elevated (>75%), i.e., a more sustained interhemispheric effect than the partial regression seen for MOp (**Fig. S4D**). In the cortico-striatal model, the week-2 rebound for SSp-ll was more pronounced than for MOp, with >80% of ipsilesional and ischemic-to-contralateral connections increasing, but all connections again declined by week 4 (**Fig. S4A,C**).

**SSp-ul** also showed a distinct pattern compared with MOp (**Fig. S5**). In the cortical stroke model, ipsilesional connectivity increased from week 1 (73%) to a peak at week 2 (100%), but this enhancement was not sustained and most connections declined by week 4 (82% decreased), in contrast to MOp where ipsilesional connectivity remained consistently elevated (>80%) across all time points (**Fig. S5A**). On the contralateral side, SSp-ul was increased at week 1 and 2, before shifting toward reduced connectivity at week 4; MOp, by comparison, showed uniformly increased contralateral connectivity at weeks 1–2 and a more mixed pattern at week 4 (**Fig. S5B**). From ischemic to contralateral, SSp-ul was mixed at weeks 1–2 and completely decreased by week 4, whereas MOp remained consistently increased (**Fig. S5C**). From contralateral to ischemic, SSp-ul again showed early increases but a stronger regression by week 4 (**Fig. S5D**). In the cortico-striatal model, SSp-ul showed a more pronounced contralateral rebound at week 2 than MOp (82% increased), but by week 4 both seeds converged to uniform connectivity loss.

**Figure S1. Representative lesion-mask registration and lesion-exclusion procedure in functional space.**

Representative examples from the cortical PT and cortico-striatal MCAO groups showing how lesion masks were generated and applied during preprocessing. For each model, the upper row shows a week 1 T2-weighted anatomical image without and with the manually delineated stroke mask overlaid in red. The lower row shows the corresponding rs-fMRI/EPI image, the atlas region after subtraction of the registered lesion mask, and the atlas overlay in functional space. Because the lesion is not reliably visible on EPI/rs-fMRI images, lesion masks were delineated on T2-weighted images and then registered to the corresponding functional images. Lesion-affected voxels were excluded from regional time-series extraction and FC computation while preserving the spatial representation of affected regions for visualization.

**Figure S2. Day 0 FC-pattern similarity between PT and MCAO cohorts.** Day 0 FC values were collapsed into unique undirected connections and normalized within each mouse to reduce the influence of global FC magnitude differences. The group-average normalized connection profiles were positively correlated between PT and MCAO, indicating broadly similar baseline FC organization despite differences in mouse strain, stroke model, and absolute baseline FC magnitude.

**Figure S3. Fully labeled FC matrices for the sensorimotor network.** Adjacency matrices showing FC changes from baseline to week 1 after stroke in the cortical (**A**) and cortico-striatal lesion model (**B**). The matrices include all 28 bilateral ROIs used in the FC analysis, corresponding to 14 anatomical regions in each hemisphere (**I** - ischemic and **C** - contralateral). Regions are grouped by hemisphere and network, including the sensorimotor cortex, thalamus, and striatum. Color values indicate the difference in absolute Fisher z-transformed functional connectivity between week 1 and baseline, Positive values indicate increased absolute FC strength relative to baseline, whereas negative values indicate decreased absolute FC strength relative to baseline.

**Figure S4. Connectivity changes in the SSp-ll network.** Relative changes in FC compared to baseline within the ischemic (A), contralateral hemisphere (B), ischemic to contralateral (C), and contralateral to ischemic connections (D). The color blue indicates a weakening of connections following stroke, while red represents a strengthening of connections relative to baseline; line weight encodes the magnitude of change (legend). Black-filled points mark connections showing a statistically significant change from baseline for that target region at that time point (linear mixed-effects model with post-hoc contrasts of Day within Region, adjusted for multiple comparisons; *P* < 0.05).

**Figure S5. Connectivity changes in the SSp-ul network.** Relative changes in FC compared to baseline within the ischemic (A) and contralateral hemisphere (B) as well as for the ischemic to contralateral (C) and contralateral to ischemic connections (D). The color blue indicates a weakening of connections following stroke, while red represents a strengthening of connections compared to baseline; line weight encodes change magnitude (legend). Black-filled points mark connections showing a statistically significant change from baseline for that target region at that time point (linear mixed-effects model with post-hoc contrasts of Day within Region, adjusted for multiple comparisons; *P* < 0.05).

**Table S1. Selected regions used for targeted FC analysis**

| **Region group** | **Abbrev.** | **Full region name** |
| --- | --- | --- |
| Primary somatosensory cortex | SSp-bfd | Primary somatosensory area, barrel field |
| Primary somatosensory cortex | SSp-ll | Primary somatosensory area, lower limb |
| Primary somatosensory cortex | SSp-m | Primary somatosensory area, mouth |
| Primary somatosensory cortex | SSp-n | Primary somatosensory area, nose |
| Primary somatosensory cortex | SSp-tr | Primary somatosensory area, trunk |
| Primary somatosensory cortex | SSp-ul | Primary somatosensory area, upper limb |
| Primary somatosensory cortex | SSp-un | Primary somatosensory area, unassigned/unidentified area |
| Supplemental somatosensory cortex | SSs | Supplemental somatosensory area |
| Motor cortex | MOp | Primary motor area |
| Motor cortex | MOs | Secondary motor area |
| Thalamus | DORpm | Dorsal thalamus, postero-medial / polymodal association-related region |
| Thalamus | DORsm | Dorsal thalamus, somatosensory-medial / sensory-motor-related region |
| Striatum | STRd | Dorsal striatum |
| Striatum | STRv | Ventral striatum |

**Table S2. Within-group longitudinal statistics for baseline-referenced FC.** Region- and hemisphere-resolved results from linear mixed-effects (LME) models assessing changes over time within each stroke group. For each group and region (ipsi-/contralesional hemisphere), model-based contrasts compare each post-stroke time point to the pre-stroke baseline and report the corresponding effect estimate, with 95% confidence interval (CI) and p-value. P-values shown are uncorrected (nominal P < 0.05); after multiple-comparisons correction (post hoc adjustment across regions/contrasts), none of the effects remained significant (P_adj_ ≥ 0.05).

| **Region** | **Hemisphere** | **Group** | **Day** | **P_value** | **CI (lower, upper)** |
| --- | --- | --- | --- | --- | --- |
| MOp | Contralateral | PT | 14 | 0.04015 | [-0.239, -0.006] |
| MOp | Ischemic_to_Contralateral | PT | 7 | 0.03861 | [-0.218, -0.006] |
| SSp-m | Contralateral | PT | 14 | 0.04079 | [-0.239, -0.005] |
| SSp-un | Contralateral | PT | 14 | 0.04156 | [-0.239, -0.005] |

**Table S3. Between-group statistics for baseline-referenced functional connectivity changes.** Region and hemisphere-resolved between-group comparisons of baseline-referenced FC change (ΔFC = FCpost − FCbaseline) at each post-stroke time point. ΔFC was analyzed using LME-based contrasts to test group differences per region and hemisphere at weeks 1, 2, and 4, and results are reported as effect estimate, 95% CI, and p-value. P-values shown are uncorrected (nominal P < 0.05); after multiple-comparisons correction, no comparisons remained significant (Padj ≥ 0.05)

| **Region** | **Hemisphere** | **Day** | **P_value** | **CI (lower, upper)** |
| --- | --- | --- | --- | --- |
| DORpm | Ischemic_to_Contralateral | 7 | 0.02338 | [0.041, 0.568] |
| MOp | Contralateral_to_Ischemic | 7 | 0.02736 | [0.032, 0.536] |
| MOp | Contralateral_to_Ischemic | 28 | 0.04676 | [0.004, 0.511] |
| MOp | Ischemic | 7 | 0.01673 | [0.051, 0.510] |
| MOp | Ischemic | 28 | 0.02405 | [0.035, 0.498] |
| MOp | Ischemic_to_Contralateral | 7 | 0.0103 | [0.081, 0.608] |
| MOp | Ischemic_to_Contralateral | 28 | 0.00783 | [0.095, 0.625] |
| MOs | Contralateral | 28 | 0.03744 | [0.018, 0.607] |
| MOs | Contralateral_to_Ischemic | 7 | 0.03933 | [0.013, 0.517] |
| MOs | Contralateral_to_Ischemic | 28 | 0.03723 | [0.016, 0.523] |
| MOs | Ischemic | 7 | 0.04007 | [0.011, 0.470] |
| SSp-ll | Contralateral_to_Ischemic | 7 | 0.0268 | [0.033, 0.537] |
| SSp-ll | Contralateral_to_Ischemic | 28 | 0.02173 | [0.043, 0.551] |
| SSp-ll | Ischemic | 7 | 0.03905 | [0.012, 0.472] |
| SSp-n | Contralateral_to_Ischemic | 7 | 0.01629 | [0.057, 0.561] |
| SSp-m | Contralateral_to_Ischemic | 7 | 0.02361 | [0.039, 0.543] |
| SSp-tr | Ischemic | 7 | 0.02182 | [0.039, 0.499] |
| SSp-tr | Ischemic_to_Contralateral | 7 | 0.01429 | [0.066, 0.593] |
| SSp-ul | Ischemic | 7 | 0.03597 | [0.016, 0.476] |
| SSp-ul | Ischemic_to_Contralateral | 7 | 0.0387 | [0.014, 0.541] |
| SSp-un | Ischemic | 7 | 0.04534 | [0.005, 0.464] |
| SSp-tr | Ischemic_to_Contralateral | 7 | 0.01429 | [0.066, 0.593] |
| SSs | Contralateral_to_Ischemic | 7 | 0.01098 | [0.075, 0.579] |
| SSs | Ischemic_to_Contralateral | 7 | 0.04566 | [0.006, 0.631] |
| STRd | Ischemic_to_Contralateral | 7 | 0.02954 | [0.029, 0.556] |
| STRd | Ischemic_to_Contralateral | 28 | 0.02404 | [0.040, 0.571] |

## References

1. Pallast N, Diedenhofen M, Blaschke S, et al. Processing Pipeline for Atlas-Based Imaging Data Analysis of Structural and Functional Mouse Brain MRI (AIDAmri). *Front Neuroinform*. 2019;13:42. doi:10.3389/fninf.2019.00042

2. Li C, Gore JC, Davatzikos C. Multiplicative intrinsic component optimization (MICO) for MRI bias field estimation and tissue segmentation. *Magnetic resonance Imagine*. 2014;32(7). doi:10.1016/j.mri.2014.03.010

3. Yushkevich PA, Piven J, Hazlett H, et al. User-guided 3D active contour segmentation of anatomical structures: Significantly improved efficiency and reliability. *NeuroImage*. 2006;31(3):1116-1128. doi:10.1016/j.neuroimage.2006.01.015
